# Synthesis, X-ray Structure, Spectroscopic Properties and DFT Studies of a Novel Schiff Base

**DOI:** 10.3390/ijms151018706

**Published:** 2014-10-17

**Authors:** Kew-Yu Chen, Hsing-Yang Tsai

**Affiliations:** Department of Chemical Engineering, Feng Chia University, Taichung 40724, Taiwan; E-Mail: p0156676@fcu.edu.tw

**Keywords:** salicylideneaniline derivatives, Schiff bases, ESICT, ESIPT, solvatochromism, Lippert-Mataga equation, Schaefer’s correlation, density functional theory calculations

## Abstract

A series of Schiff bases, salicylideneaniline derivatives **1**–**4**, was synthesized under mild conditions and characterized by ^1^H NMR, HRMS, UV-Vis and fluorescence spectra, and single-crystal X-ray diffraction. In solid and aprotic solvents **1**–**4** exist mainly as *E* conformers that possess an intramolecular six-membered-ring hydrogen bond. A weak intramolecular C–H···F hydrogen bond is also observed in fluoro-functionalized Schiff base **4**, which generates another S(6) ring motif. The C–H···F hydrogen bond further stabilizes its structure and leads it to form a planar configuration. Compounds **1**–**3** exhibit solely a long-wavelength proton-transfer tautomer emission, while dipole-functionalized Schiff base **4** shows remarkable dual emission originated from the excited-state intramolecular charge transfer (ESICT) and excited-state intramolecular proton transfer (ESIPT) states. Furthermore, the geometric structures, frontier molecular orbitals (MOs) and the potential energy curves for **1**–**4** in the ground and the first singlet excited state were fully rationalized by density functional theory (DFT) and time-dependent DFT calculations.

## 1. Introduction

**Figure 1:**
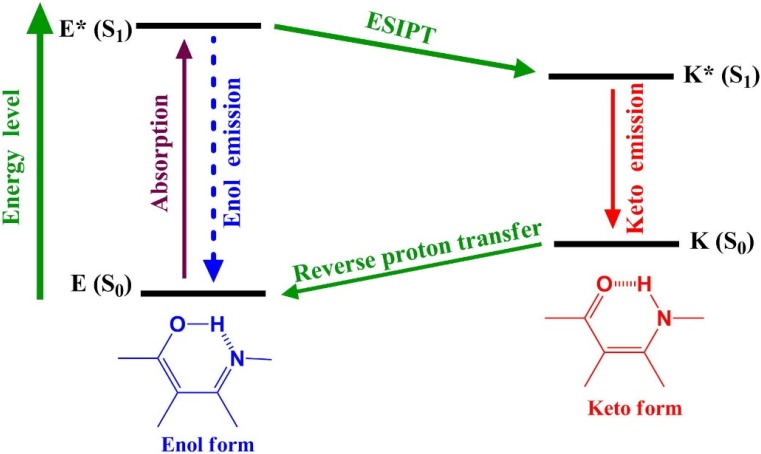
Characteristic four-level photocycle scheme of the ESIPT process.

**Figure 2:**
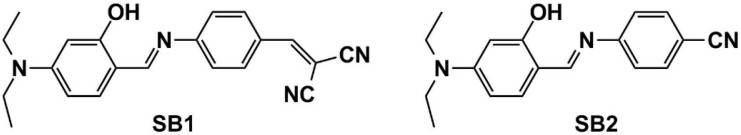
The structures of dipole-functionalized salicylideneaniline derivatives **SB1** and **SB2**.

**Scheme 1:**
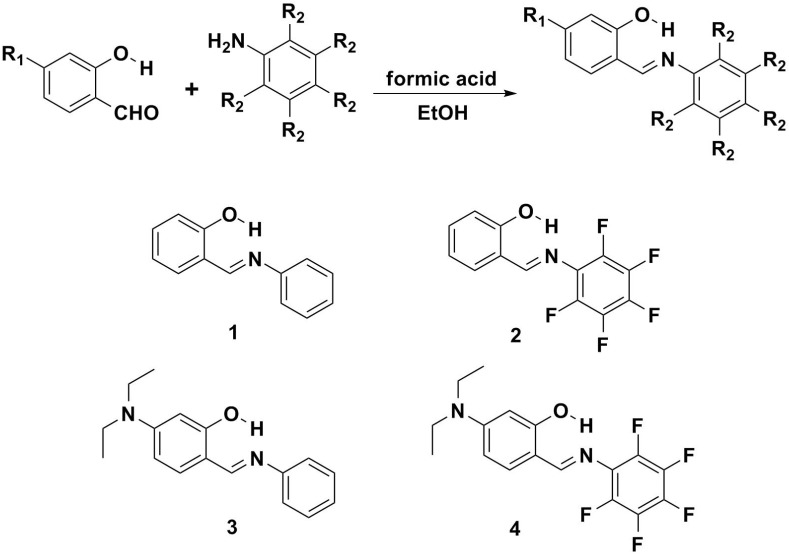
The synthetic route and the structures for **1**–**4**.

Schiff bases, named after Hugo Schiff, formed by a condensation reaction of a primary amine with an aldehyde or ketone were reported in the 19th century [1]. Structurally, a Schiff base is a nitrogen analogue of an aldehyde or ketone in which the carbonyl group is replaced by an imine group [2,3,4,5]. Since then a number of methods for the synthesis of imines have been described [6,7,8,9]. Schiff bases have been drawing considerable attention due to their potential application in many areas such as liquid crystals [10,11,12,13], organic dyes [14,15,16,17,18,19,20,21], catalysts [22,23,24], and intermediates in organic synthesis [25,26,27,28,29,30]. Moreover, many Schiff bases exhibit a broad range of biological activities [31,32,33,34,35]. For example, salicylideneaniline (**1**, Scheme 1) derivatives are effective against *Mycobacterium tuberculosis* H37Rv [36]. The excited-state intramolecular proton transfer (ESIPT) reaction [37,38,39,40,41,42,43,44,45,46,47,48,49,50,51,52,53,54] of salicylideneaniline derivatives, which incorporates transfer of a hydroxyl proton to the imine nitrogen through a pre-existing intramolecular hydrogen bonding configuration, has also been extensively investigated in the literature [55,56]. As shown in Figure 1, the resulting proton-transfer tautomer (keto-form) exhibits significant differences in structure and electronic configuration from its corresponding ground state, *i.e.*, a large Stokes shifted S_1_ (K *)→S_0_ (K) fluorescence. This unusual optical property has found many important applications, including probes for solvation dynamics [57] and biological environments [58], photochromic materials [59], chemosensors [60,61,62,63,64], and organic light-emitting diodes [65,66,67]. In addition, many relevant examples have recently been examined by anchoring the ESIPT molecules with a strong electron-donating group (dialkylamino group), so that upon Franck-Condon excitation, excited-state intramolecular charge transfer (ESICT) may take place [68]. In an effort to expand the scope of salicylideneaniline-based chromophores available for designing systems for molecular recognition [69] and ESICT/ESIPT coupled reaction [70], we have recently synthesized two dipole-functionalized salicylideneaniline derivatives **SB1** and **SB2**, respectively (Figure 2). Herein, we report a novel dipole-functionalized salicylideneaniline derivative (**4**) that shows remarkable dual emission originated from the ESICT and ESIPT states.

## 2. Results and Discussion

### 2.1. Synthesis

The synthetic route and the structures of compounds **1**–**4** are shown in Scheme 1. These Schiff bases were easily prepared through condensation reactions between nonsubstituted/substituted salicylic aldehydes and nonsubstituted/substituted anilines [71]. Detailed synthetic procedures and product characterization are provided in the Experimental Section. 

### 2.2. Hydrogen Bond Studies

The dominance of an enol-form for **1**–**4**, namely intramolecular hydrogen-bond formation between O–H and N, is well supported by a combination of ^1^H NMR and X-ray single-crystal analyses [72,73]. In the ^1^H NMR studies, the existence of a strong hydrogen bond between O–H and N is evidenced by the observation of a large downfield shift of the proton peak at δ > 12 ppm for all salicylideneaniline derivatives **1**–**4**, the values of which are in the order of **3** (13.85 ppm) > **1** (13.26 ppm) > **4** (12.74 ppm) > **2** (12.12 ppm) in dry CDCl_3_ (Table 1). The hydrogen bonding energy (∆*E* in kcal/mol) of **1**–**4** can be calculated by introducing Schaefer’s correlation [74], expressed as ∆δ = (−0.4 ± 0.2) + ∆*E*, where ∆δ is given in parts per million for the difference between chemical shift in the O–H peak of **1**–**4** and that in phenol (δ 4.29). The hydrogen-bonding energy is therefore estimated to be **3** (9.96 ± 0.2 kcal/mol) > **1** (9.37 ± 0.2 kcal/mol) > **4** (8.85 ± 0.2 kcal/mol) > **2** (8.23 ± 0.2 kcal/mol), which is consistent with the theoretical calculations (Table 1 and Supplementary Materials). A decrease in the hydrogen bonding strength upon an increase in the electron-withdrawing fluoro substituent at the phenyl ring is observed. Note, that the substitution of the hydrogen atoms at the phenyl ring in **3** by electron-withdrawing fluoro substituents, forming **4**, seems to decrease the basicity of the imine through an inductive effect. As a result, compound **4** exhibits a small upfield shift of the hydroxyl proton, and hence, a weaker hydrogen bond relative to **3**. 

**Table 1:** Calculated and experimental parameters for **1**–**4**.

Compound	^1^H NMR ^a^	HB length ^b^	*E*_HB_^c^	*E*_HB_^d^
**1**	13.26	2.641	9.37	13.79
**2**	12.12	2.648	8.23	12.27
**3**	13.85	2.638	9.96	14.35
**4**	12.74	2.643	8.85	12.50

^a^ The hydroxy proton signals (in ppm); ^b^ The intramolecular hydrogen bond length (O···N(1)) obtained from our DFT calculation (in Å); ^c^ The intramolecular hydrogen bonding obtained from Schaefer’s correlation (in kcal/mol); ^d^ The intramolecular hydrogen bonding obtained from DFT calculation (in kcal/mol).

### 2.3. X-ray Structure

**Figure 3:**
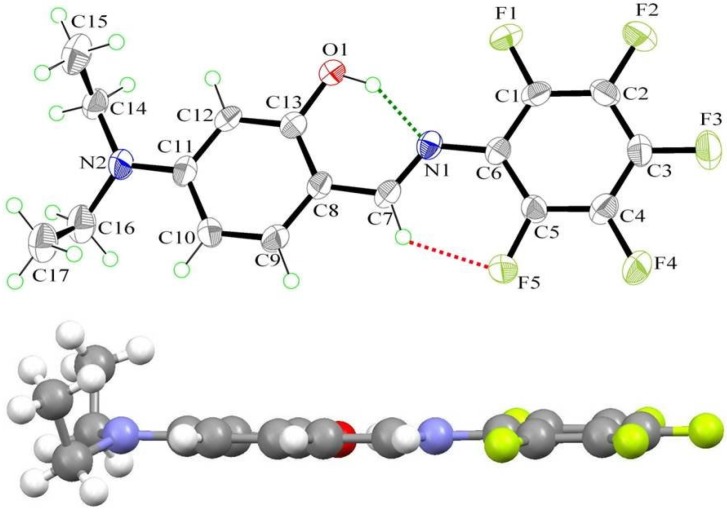
The molecular structure of **4**, showing the atom-labelling scheme. Displacement ellipsoids are drawn at the 50% probability level. Green and red dashed lines denote the intramolecular O–H···N and C–H···F hydrogen bonds, respectively.

**Figure 4:**
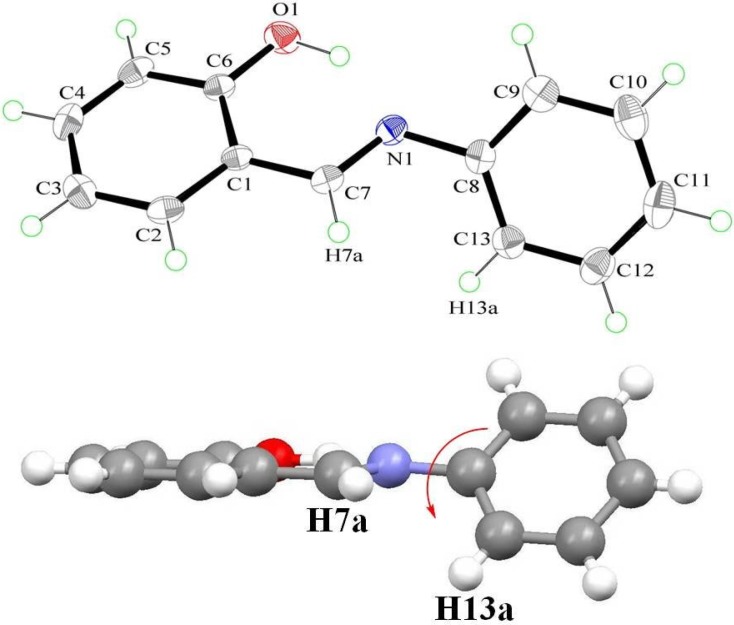
The molecular structure of **1** [75,76], showing the atom-labelling scheme. Displacement ellipsoids are drawn at the 50% probability level.

The structure of **4** was further confirmed by single-crystal X-ray diffraction analysis (Figure 3). It crystallizes in the monoclinic space group *P*2_1_/c, with *a* = 7.6613(9), *b* = 21.251(3), *c* = 9.6820(13) Å, α = 90°, β = 95.386(4)°, γ = 90°. The complete molecule is nearly planar (except for diethylamino group), as indicated by the key torsion angles (Table 2). The maximum deviations from the mean plane through the non-H atoms are 0.100(2) Å for atom O(1) and 0.067(2) Å for atom C(1). The dihedral angle between the mean planes of the phenol ring and phenyl ring is 4.2(2)°, which is smaller than that [75,76] of **1** (Figure 4). Additionally, compound **4** possesses a strong intramolecular O(1)–H···N(1) hydrogen bond that generates an S(6) ring motif [77,78]. The dihedral angle between the mean planes of the S(6) ring and the phenol ring is 3.36(2)°. This, together with the 2.626(3) Å of O(1)···N(1) distance and 152(4)° of O(1)–H···N(1), strongly supports the S(6) ring formation. The distance between O(1) and N(1) along the O(1)–H···N(1) hydrogen bond is in the order of **4** (2.626 Å) > **1** (2.615 Å) > **3** (2.572 Å) and consistent with the theoretical calculations.

**Table 2:** Comparison of the experimental and optimized geometric parameters of **4** (Å and °).

	X-ray	DFT
Bond lengths (Å)		
O–C(13)	1.357(4)	1.341
C(1)–C(2)	1.372(4)	1.389
C(5)–C(6)	1.398(4)	1.409
N(1)–C(6)	1.400(4)	1.386
N(1)–C(7)	1.299(4)	1.305
N(2)–C(11)	1.368(4)	1.379
C(12)–C(13)	1.379(4)	1.393
C(5)–F(5)	1.342(3)	1.348
Bond angles (°)		
O(1)–C(13)–C(12)	117.6(3)	117.8
C(2)–C(3)–C(4)	118.2(3)	119.2
C(8)–C(7)–N(1)	121.7(2)	121.3
C(1)–C(6)–N(1)	117.4(1)	116.3
C(11)–C(12)–C(13)	121.0(3)	121.7
C(4)–C(5)–F(5)	116.5(3)	117.0
Torsion angles (°)		
O(1)–C(13)–C(8)–C(7)	2.8(2)	0.3
N(1)–C(6)–C(1)–C(2)	178.8(2)	178.6
N(2)–C(11)–C(10)–C(9)	179.7(2)	178.1
C(8)–C(7)–N(1)–C(6)	179.2(2)	179.5

**Figure 5:**
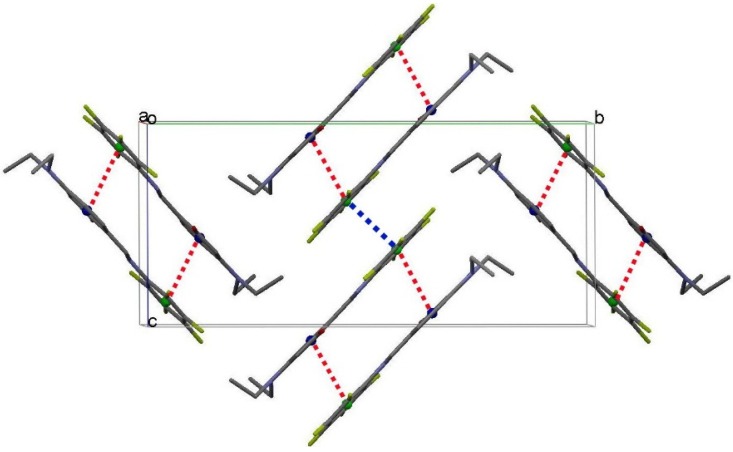
A stereoview of part of the crystal structure of **4**, viewed along the *a*-axis (all hydrogen atoms are omitted for clarity). Red and blue dashed lines denote the intermolecular π···π interactions. *C*g1 (blue circles) and *C*g2 (green circles) are the centroids of the C8–C13 and C1–C6 rings, respectively.

There is another weak intramolecular C–H···F hydrogen bond in **4** (2.802(2) Å of C(7)···F(5) distance and 129° of C(7)–H(7)–F(5)) that forms another S(6) ring motif (red dash line in Figure 3). The C–H···F hydrogen bond further stabilizes its structure and leads it to form a planar configuration. Furthermore, intermolecular π–π stacking is also observed in the crystal structure, which links a pair of molecules into a cyclic centrosymmetric dimer (Figure 5). Pertinent measurements for these π···π interactions are: centroid–centroid distances of 3.585(3) (red dashed lines, symmetry code: 1–x, –y, 1–z) and 4.151(2) Å (blue dashed line, symmetry code: 1–x, –y, –z). The closest contact distances are C(6)···C(13) of 3.269(4) Å (symmetry code: 1–x, –y, 1–z) and C(2)···C(11) of 3.424(5) Å (symmetry code: 1–x, –y, 1–z).

### 2.4. Optical Properties

**Figure 6:**
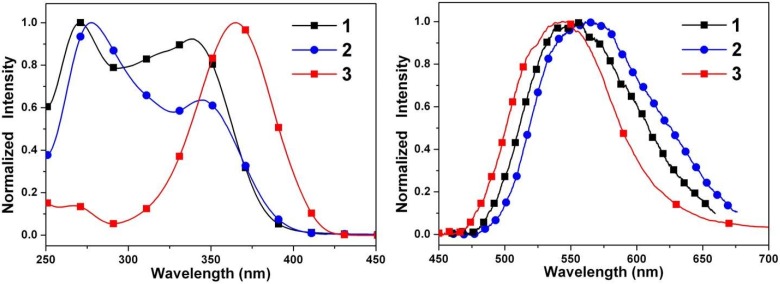
Normalized absorption (**left**) and emission (**right**) spectra of **1**–**3** in cyclohexane.

Figure 6 shows the steady-state absorption and emission spectra of **1**–**3** in cyclohexane. Compounds **1**–**3** show a S_0_→S_1_ electronic transition at about 350 nm. Careful examination of the absorption spectra of **1**–**3** also shows that the longest wavelength absorption band of both **2** and **3** is slightly red-shifted relative to that of **1**. The decrease in the energy band gap is attributed to either a decrease in the LUMO energy level or an increase in the HOMO energy level (vide infra). As for the steady-state emission, **1**–**3** all exhibit solely a long-wavelength fluorescence (>540 nm) in cyclohexane. Figure 6 also displays a large separation of the energy gap between the 0–0 onset of the absorption and emission. The Stokes shift of the emission, defined by peak (absorption)-to-peak (emission) gap in terms of frequency, is calculated to be >9100 cm^−1^ for **1**–**3** (Table 3). Accordingly, the assignment of 547–565 nm fluorescence for **1**–**3** in cyclohexane to a proton-transfer tautomer emission is unambiguous, and ESIPT takes place from the phenolic proton O–H to the imine nitrogen, forming the keto tautomeric species.

**Figure 7:**
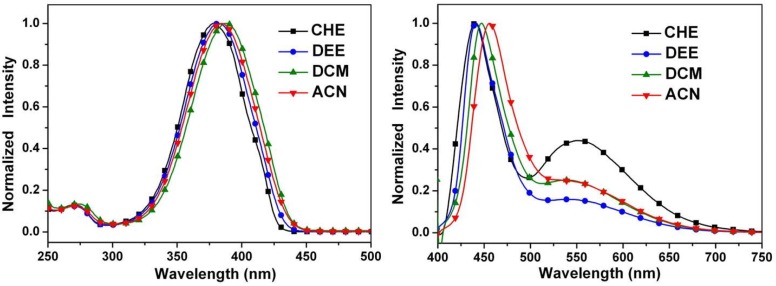
Normalized absorption (**left**) and emission (**right**) spectra of **4** in cyclohexane (CHE), diethyl ether (DEE), dichloromethane (DCM), and acetonitrile (ACN).

Figure 7 depicts the absorption and emission spectra of **4** in solvents of varying polarity. It is apparent that the absorption spectrum of **4** is a close resemblance to that of **3**. Despite the similarity in absorption spectra, in which the S_0_→S_1_ peak wavelengths are both located at 365–379 nm for **3** and **4**, the corresponding fluorescence shows remarkable differences. In contrast to a unique proton-transfer emission in **3**, dual emission maximized at 436 and 551 nm was observed for **4** in cyclohexane. The 551 nm band can clearly be assigned to the keto emission resulting from ESIPT, while the 436 nm band is absent in **3** and is accordingly ascribed to the fluorescence of the enol species.

As shown in Figure 7, similar dual emission was observed for **4** in polar solvents. As the solvent polarity increases, however, the intensity ratio for the keto-form *versus* enol-form intensity decreases. While the peak wavelength of the keto-form fluorescence remains unchanged, the enol-form fluorescence of **4** reveals intelligible solvent-polarity dependence, being shifted from 436 nm in cyclohexane to 456 nm in acetonitrile. The emission spectra shift significantly to the red upon increasing solvent polarity, indicating an intramolecular charge transfer (ICT) characteristic for the excited state of the compound. In view of the low ionization potential of the diethylamino substituent and the high electron affinity of the pentafluorophenyl group, the occurrence of ESICT in **4** is expected. Using the fluorescence solvatochromic shift method [79,80,81], we measure the stabilization of the excited states of **4**. The change of magnitudes for dipole moments between ground and excited states, *i.e.*, $\Delta \mu =|\vec{\mu_{e}}-\vec{\mu_{g}}|$, can be estimated by the Lippert–Mataga equation and expressed as:

(1) $${\bar{\upsilon }}_{a}-{\bar{\upsilon }}_{f}=\frac{2}{hc}{\left(\mu_{e}-\mu_{g}\right)}^{2}{a_{0}}^{-3}\Delta f+const.$$

where *h* is the Planck constant, *c* is the speed of light, and $a_{0}$ denotes the cavity radius in which the solute resides, calculated to be 5.3 Å via Hartree–Fock theories with 6-31G ** basis, ${\bar{\upsilon }}_{a}-{\bar{\upsilon }}_{f}$ is the Stokes shift of the absorption and fluorescence peak maximum, and Δf is the orientation polarizability defined as:

(2) $$\Delta f=f\left(\varepsilon \right)-f\left(n^{2}\right)=\frac{\varepsilon -1}{2\varepsilon +1}-\frac{n^{2}-1}{2n^{2}+1}$$

**Figure 8:**
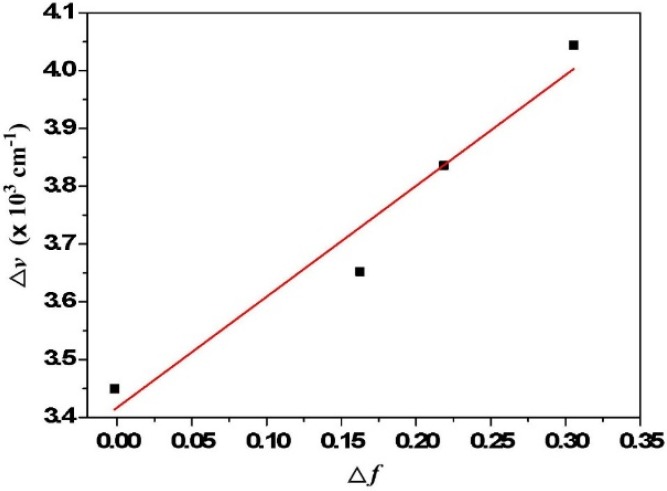
Lippert-Mataga plot for **4**. The solvents from left to right are: (1) cyclohexane; (2) diethyl ether; (3) dichloromethane; (4) acetonitrile.

The plot of the Stokes shift ${\bar{\upsilon }}_{a}-{\bar{\upsilon }}_{f}$ as a function of Δf is nearly linear for **4** (Figure 8), and $\Delta \mu =|\vec{\mu_{e}}-\vec{\mu_{g}}|$ can be calculated as 5.0 D. 

**Table 3:** Summary of optical absorption and emission properties of **1**–**4** in cyclohexane.

Compound	λ_abs_ (nm) ^a^	λ_em_ (nm) ^a^	Stokes shift ^b^	Φ ^c^ × 10^3^
**1**	339	556	11,513	3.5
**2**	345	565	11,286	5.2
**3**	365	547	9116	8.5
**4**	379	436, 551	34,498,236	19.6

^a^ Measured at 2×10^−5^ M; ^b^ In cm^−^^1^; ^c^ Quantum yield (determined with compound **1**).

### 2.5. Quantum Chemistry Computation

For deeper insight into the molecular structures and electronic properties of **1**–**4**, quantum mechanical calculations were performed using density functional theory (DFT) at the B3LYP/6-31G** level. The values of bond lengths, bond angles, and torsion angles for Schiff base **4** were compared with its crystal structural values. Table 2 compares the experimental and optimized geometric parameters of **4**. One can see that there are no substantial differences between the experimental and DFT/B3LYP data. The results demonstrate that the optimized geometry can effectively reproduce the crystal structure. 

**Figure 9:**
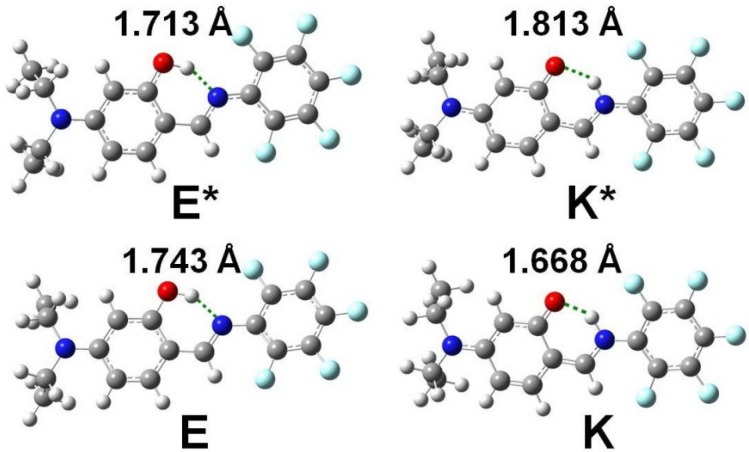
The optimized geometric structures of enol (E) and keto (K) form for **4** in the ground (S_0_) and the first singlet excited state (S_1_) together with the intramolecular hydrogen bond lengths.

Figure 9 shows the optimized geometric structures and the corresponding hydrogen bond lengths of enol and keto form for **4** in the ground (S_0_) and the first singlet excited state (S_1_). From E (K *) to E * (K), the intramolecular hydrogen bond length decreases from 1.743 (1.813) Å to 1.713 (1.668) Å. The results give the evidence for the strengthening of the intramolecular hydrogen bond from S_0_→S_1_ (S_1_→S_0_). Therefore, there is no doubt that the decrease of intramolecular hydrogen bond lengths from E (K *) to E * (K) is a principal positive factor for the ESIPT (GSIPT: ground state intramolecular proton transfer) reaction. 

**Figure 10:**
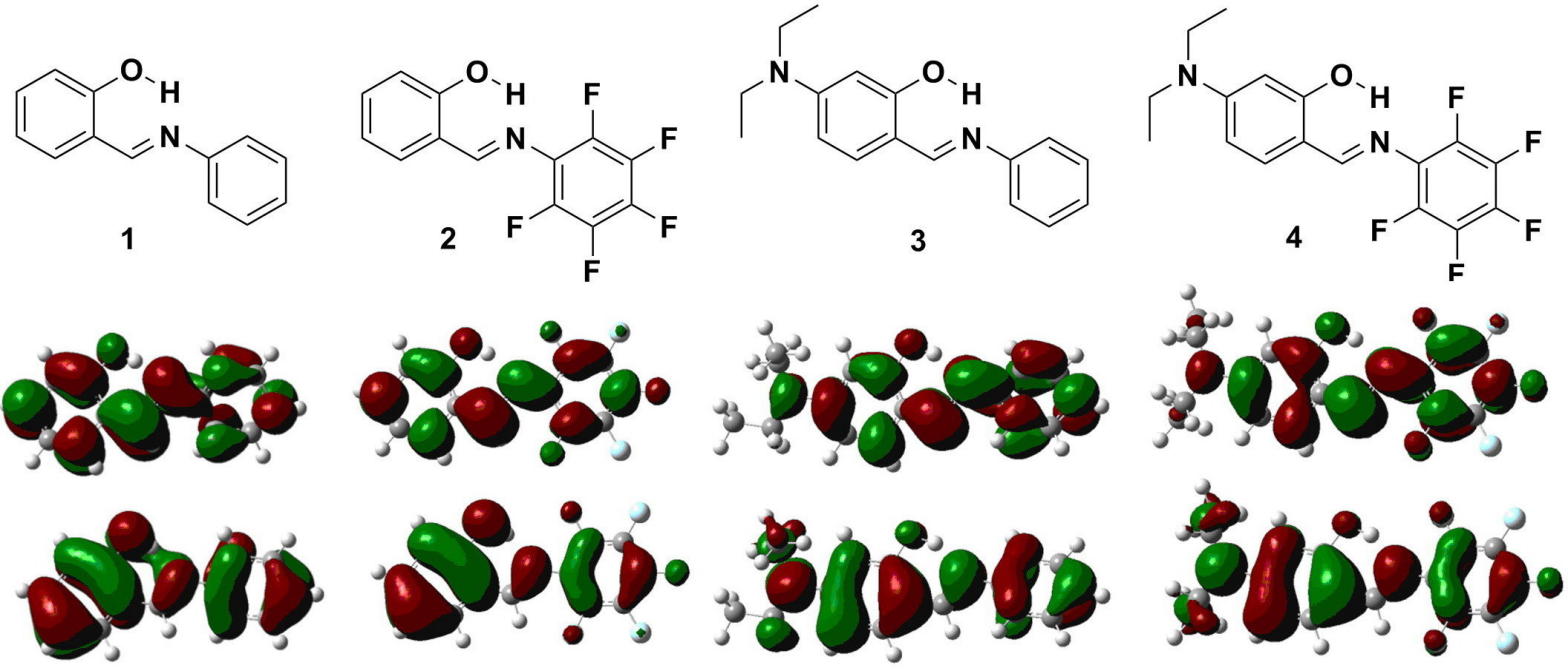
Calculated frontier orbitals for **1**–**4**. The upper graphs are the LUMOs and the lower ones are the HOMOs.

**Figure 11:**
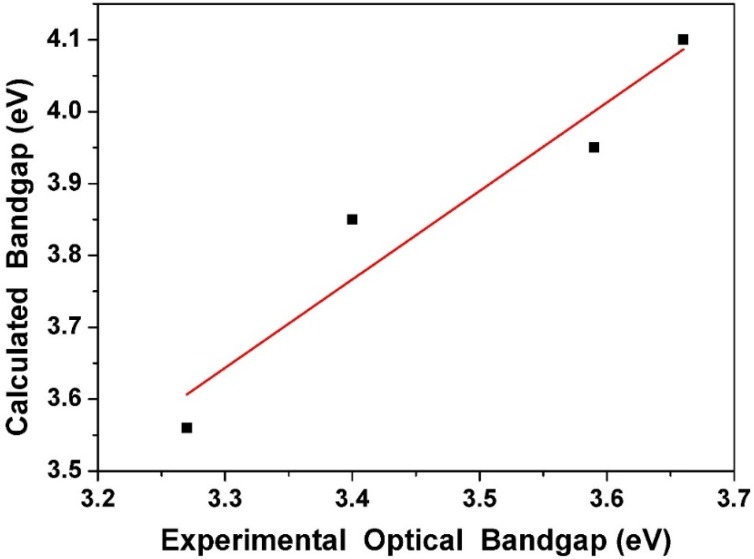
Plot of calculated optical bandgap (eV) in the gas phase *νs.* experimental optical bandgap in solution.

The highest occupied molecular orbitals (HOMOs) and the lowest unoccupied molecular orbitals (LUMOs) of **1**–**4** are shown in Figure 10. Both HOMO and LUMO in **1**–**3** are delocalized extensively over the whole π-conjugated system. In contrast, the HOMO of **4** is delocalized mainly on the diethylamino group and the phenol ring, while the LUMO is extended from the imine group to the phenyl ring. The result supports the viewpoint of ICT in **4** from diethylamine to the electron-deficient pentafluorophenyl ring. The calculated HOMO/LUMO energy levels of **4** are –5.32/–1.76 eV (Table 4), respectively, and those of **3** are –5.10/–1.25, respectively, yet both are higher than those of **1** (–6.16/–2.06 eV). In contrast, both the HOMO/LUMO (–6.21/–2.26) energy levels of **2** are lower than those of **4**. This can be explained by the fact that the pentafluorophenyl substituent is an electron-withdrawing group and hence decreases both the HOMO and LUMO energy levels. An opposite effect is observed when introducing an electron-donating diethylamino group into the phenol ring. The trend of the HOMO-LUMO energy gap is **1** > **2** > **3** > **4**, which is in good agreement with the experimental data. Additionally, the comparison of experimental optical absorption bandgaps and calculated results is shown in Figure 11. It is noteworthy that the linear plotted line gives a sufficiently straight feature (*r* = 0.96119, SD = 0.07698). Consequently, the DFT calculations provide reasonable explanations for their electronic structure and absorption spectra.

**Table 4:** Calculated and experimental parameters for **1**–**4**.

Compound	HOMO ^a^	LUMO ^a^	E_g_^a^	E_g_^b^
**1**	−6.16	−2.06	4.10	3.66
**2**	−6.21	−2.26	3.95	3.59
**3**	−5.10	−1.25	3.85	3.40
**4**	−5.32	−1.76	3.56	3.27

^a^ Calculated by DFT/B3LYP (in eV); ^b^ At absorption maxima (*E*_g_ = 1240/λ_max_, in eV).

**Figure 12:**
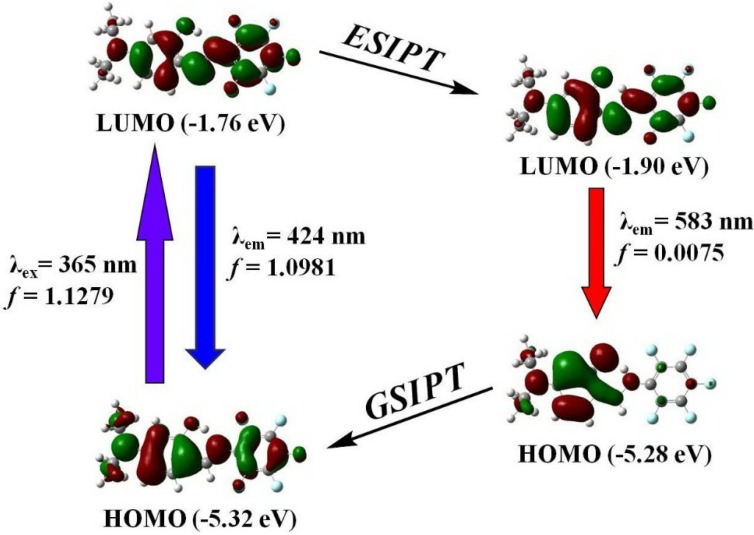
Selected frontier molecular orbitals involved in the excitation and emission of **4**.

Figure 12 shows the HOMO and LUMO of enol and keto form of **4**. Upon the photoexcitation, the electron density formerly located on the diethylamino group decreases while that on the pentafluorophenyl ring increases, which indicates that the excitation from E to E * should involve intramolecular electron density transfer from amino group to pentafluorophenyl ring. The energy level of the LUMO decreases from −1.76 to −1.90 eV with completion of the ESIPT reaction, indicating that it is thermodynamically favorable enough to drive the production of the excited-state keto tautomer. Moreover, the calculated enol (424 nm) and keto (583 nm) emissions are reasonably close to the experimental data. It can be also observed that the first excited states of **1**–**4** for both enol and keto forms are a dominant π→π* transition from the HOMO to the LUMO (Table 5).

**Figure 13:**
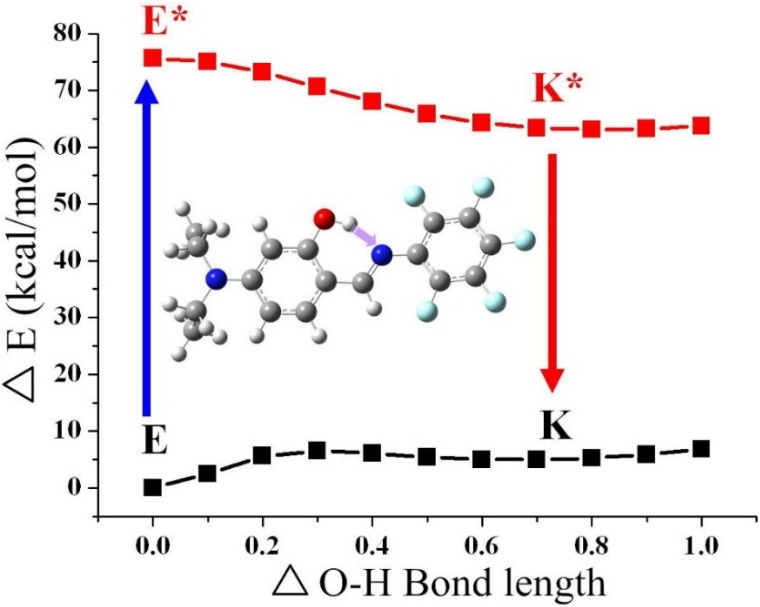
Potential energy curves from enol form to keto form of **4** at the ground state and excited state. The calculations are based on the optimized ground state geometry (S_0_ state) at the B3LYP/6-31G **/level.

In an effort to explain the ESIPT properties of compounds **1**–**4**, the potential energy curves of the intramolecular proton transfer (*i.e.*, the transformation from the enol form to the keto form) at both the ground state and the excited state were studied (Figure 13 and Table 6). The full geometry optimization based on the B3LYP/6-31G ** theoretical level reveals that the enol form of **1**, **2**, **3**, and **4** in the ground state is more stable than the corresponding proton-transfer tautomer by 4.4, 5.9, 6.1, and 5.5 kcal/mol, respectively. Further calculations show that the corresponding proton-transfer tautomer is lower in energy than the respective **1**, **2**, **3**, and **4** by 11.6, 11.8, 12.5, and 12.0 kcal/mol, respectively, in the excited state. The results clearly indicate that ESIPT for all Schiff bases **1**–**4** is thermodynamically favorable, which is consistent with the experimental results.

**Table 5:** Selected electronic excitation energies and corresponding oscillator strengths (*f*), main configurations, and CI coefficients of the low-lying electronically excited states of compounds **1**–**4** ^a^.

Compound	Singlet	Electronic Transition	Energy	*f*	Composition ^b^	CI ^c^
**1**	UV–vis	S_0_→S_1_	3.67 eV/338 nm	0.3302	H→L	0.68789
	FL	S_1_→S_0_	2.28 eV/542 nm	0.1229	H→L	0.68020
**2**	UV–vis	S_0_→S_1_	3.59 eV/345 nm	0.2603	H→L	0.68034
	FL	S_1_→S_0_	2.38 eV/521 nm	0.1482	H→L	0.70535
**3**	UV–vis	S_0_→S_1_	3.44 eV/360 nm	1.0334	H→L	0.69373
	FL	S_1_→S_0_	2.18 eV/569 nm	0.0112	H→L	0.70469
**4**	UV–vis	S_0_→S_1_	3.39 eV/365 nm	1.1279	H→L	0.70201
	FL (Enol)	S_1_→S_0_	2.92 eV/424 nm	1.0981	H→L	0.70097
	FL (Keto)	S_1_→S_0_	2.12 eV/583 nm	0.0075	H→L	0.70576

^a^ Calculated by TDDFT/B3LYP/6-31G **. FL stands for fluorescence; ^b^ H stands for HOMO and L stands for LUMO. Only the main configurations are presented; ^c^ CI expansion coefficient for given excitation.

**Table 6:** Free energy changes for the transformations from enol-form to keto-form of the compounds at the ground state and excited state.

Compound	∆*E* (kcal·mol^−^^1^) ^a^	∆*E* (kcal·mol^−^^1^) ^b^
**1**	4.4	−11.6
**2**	5.9	−11.8
**3**	6.1	−12.5
**4**	5.5	−12.0

^a^ At S_0_ state (ground state); ^b^ At S_1_ state (excited state).

## 3. Experimental Section

### 3.1. General

The starting materials such as 2-hydroxybenzaldehyde, 4-(diethylamino)-2-hydroxybenzaldehyde, aniline, 2,3,4,5,6-pentafluoroaniline, formic acid, and ethanol were purchased from Merck (Whitehouse Station, NJ, USA), ACROS (Pittsburgh, PA, USA) and Sigma-Aldrich (St. Louis, MO, USA). Solvents were distilled freshly according to standard procedure. Column chromatography was performed using silica gel Merck Kieselgel *si* 60 (40–63 mesh). ^1^H NMR spectra were recorded in CDCl_3_ on a Bruker 400 MHz NMR spectrometer (Palo Alto, CA, USA). Mass spectra were recorded on a VG70-250S mass spectrometer (Tokyo, Japan). The absorption and emission spectra were measured using a Jasco V-570 UV–Vis spectrophotometer (Tokyo, Japan) and a Hitachi F-7000 fluorescence spectrophotometer (Tokyo, Japan), respectively. 

### 3.2. Synthesis

The general procedure for the synthesis of Schiff bases (**1**–**4**): Nonsubstituted/substituted aniline (6.0 mmol) and formic acid (0.1 mL) were added to a mixture of nonsubstituted/substituted 2-hydroxybenzaldehyde (5.2 mmol) and molecular sieves 4Å (0.5 g) in ethanol (25 mL) at room temperature. The mixture was refluxed for 10 h. After cooling, the mixture was poured into the cold water and extracted with CH_2_Cl_2_ and dried with anhydrous MgSO_4_. After solvent was removed, the crude product was purified by silica gel column chromatography with eluent CH_2_Cl_2_ to produce the title compounds (**1**–**4**) in 80% yield. Selected data: **1**: ^1^H NMR (400 MHz, CDCl_3_) δ 13.26 (br, 1H), 8.62 (s, 1H), 7.36~7.44 (m, 4H), 7.30~7.25 (m, 3H), 7.04 (d, *J* = 8.5 Hz, 1H), 6.96 (t, *J* = 7.5 Hz, 1H); MS (FAB) *m*/*z* (relative intensity) 198 (M + H^+^, 100); HRMS calcd. for C_13_H_12_NO 198.0919, found 198.0915. Selected data for **2**: ^1^H NMR (400 MHz, CDCl_3_) δ 12.12 (br, 1H), 8.81 (s, 1H), 7.33 (dd, *J*_1_ = 8.0 Hz, *J*_2_ = 2.0 Hz, 1H), 7.27 (m, 1H), 7.03 (dd, *J*_1_ = 8.0 Hz, *J*_2_ = 1.6 Hz, 1H), 6.64 (d, *J* = 8.0 Hz, 1H); MS (FAB) *m*/*z* (relative intensity) 288 (M + H^+^, 100); HRMS calcd. for C_1__3_H_7_F_5_NO 288.0448, found 288.0446. Selected data for **3**: ^1^H NMR (400 MHz, CDCl_3_) δ 13.85 (br, 1H), 8.42 (s, 1H), 7.15~7.40 (m, 6H), 6.26 (dd, *J*_1_ = 8.5 Hz, *J*_2_ = 2.0 Hz, 1H), 6.19 (s, 1H), 3.42 (q, *J*_1_ = 7.0 Hz, 4H), 1.23 (t, *J* = 7.0 Hz, 6H); MS (FAB) *m*/*z* (relative intensity) 269 (M + H^+^, 100); HRMS calcd. for C_17_H_21_N_2_O 269.1654, found 269.1658. Selected data for **4**: mp 119~120 °C; ^1^H NMR (400 MHz, CDCl_3_) δ 12.74 (br, 1H), 8.55 (s, 1H), 7.13 (d, *J* = 8.0 Hz, 1H), 6.26 (d, *J* = 8.0 Hz, 1H), 6.16 (s, 1H), 3.42 (q, *J* = 5.6 Hz, 4H), 1.21 (t, *J* = 5.6 Hz, 6H); ^13^C NMR (100 MHz, CDCl_3_) δ 167.64, 163.84, 152.88, 142.03, 140.04, 138.68, 136.83, 134.75, 108.81, 104.30, 97.58, 44.67, 12.58; MS (FAB) *m*/*z* (relative intensity) 359 (M + H^+^, 100); HRMS calcd. for C_1__7_H_16_F_5_N_2_O 359.1183, found 359.1189.

### 3.3. Crystal Structural Determination

A single crystal of **4** with dimensions of 0.48 mm × 0.11 mm × 0.03 mm was selected. The lattice constants and diffraction intensities were measured with a Bruker Smart 1000 CCD area detector radiation (*λ* = 0.71073 Å) at 150(2) K. An *ω*-2*θ* scan mode was used for data collection in the range of 2.32 ≤ *θ* ≤ 26.37. A total of 18,286 reflections were collected and 3203 were independent (*R*_int_ = 0.0710), of which 1957 were considered to be observed with *I* > 2*σ*(*I*) and used in the succeeding refinement. The structure was solved by direct methods with SHELXS-97 [82] and refined on *F*^2^ by full-matrix least-squares procedure with Bruker SHELXL-97 packing [83]. All non-hydrogen atoms were refined with anisotropic thermal parameters. The hydrogen atoms were refined with riding model, except for hydroxyl hydrogen, which was located from the difference Fourier map. At the final cycle of refinement, *R* = 0.0583 and *wR* = 0.1467 (*w* = 1/[*σ*^2^(*F_o_*^2^) + (0.0853*P*)^2^ + 0.6935*P*], where *P* = (*F_o_*^2^ + 2*F_c_*^2^)/3), *S* = 1.072, (Δ/*σ*)_max_ = 0.001, (Δ/*ρ*)_max_ = 0.243 and (Δ/*ρ*)_min_ = −0.253 e/Å^3^. Crystallographic data for compound **4** have been deposited with the Cambridge Crystallographic Data Center as supplementary publication number CCDC 1023665 [84].

### 3.4. Computational Methods

All the electronic structure calculations were carried out using the Gaussian 03 program [85]. All the geometry optimizations for compounds **1**–**4** in the ground and the first excited states were performed using density functional theory (DFT) and time-dependent density functional theory (TDDFT), respectively. The hybrid DFT functional B3LYP has proven to be a suitable DFT functional to describe hydrogen bond [86]. Vibrational frequencies were also performed to check whether the optimized geometrical structures for all compounds were at energy minima, transition states, or higher order saddle points. After obtaining the converged geometries, the TD-B3LYP/6-31G ** was used to calculate the vertical excitation energies. Emission energies were obtained from TDDFT/B3LYP/6-31G ** calculations performed on S_1_ optimized geometries. The phenomenon of photo-induced proton transfer (PT) reaction in **1**–**4** can be most critically addressed and assessed by evaluating the potential energy curve (PEC) for the PT reaction. For the S_0_ state all of the other degrees of freedom are relaxed without imposing any symmetry constraints. The excited-state (S_1_) PEC for the ESIPT reaction in **1**–**4** has been constructed on the basis of TDDFT optimization method. The energy shown in the curves are relative values, with the lowest point on the curve as zero.

## 4. Conclusions

A novel dipole-functionalized Schiff base **4**, as well as three structure-similar derivatives **1**–**3**, has been synthesized under mild conditions and characterized by ^1^H NMR, HRMS spectra, and single-crystal X-ray diffraction. In solid and aprotic solvents **1**–**4** exist mainly as *E* conformers that possess a strong intramolecular hydrogen bond. Compounds **1**–**3** exhibit solely a long-wavelength proton-transfer tautomer emission, while dipole-functionalized salicylideneaniline **4** shows remarkable dual emission originated from the ESICT and ESIPT states. Analysis of the geometric structures clearly demonstrates that the intramolecular hydrogen bond length is shortened upon photoexcitation, which is regarded as a crucial factor for ESIPT. The results make further rational design of the ESICT/ESIPT coupled systems feasible by modifying the ESIPT molecules.

## Supplementary Materials

Supplementary materials can be found at http://www.mdpi.com/1422-0067/15/10/18706/s1.

## Supplementary Files

Supplementary File 1
