# Supplementary material for: Synthesis, X-ray Structure, Spectroscopic Properties and DFT Studies of a Novel Schiff Base
Source: Int J Mol Sci. 2014 Oct 17;15(10):18706–24. doi: 10.3390/ijms151018706 (PMC4227241; doi:10.3390/ijms151018706)
Supplement: Supplementary File 1 [file ijms-15-18706-s001.pdf]

Supplementary Information

Figure S1. Computed energies of different conformers of **1** are specified relative to the Enol-form (DFT/B3LYP/6-31G **).

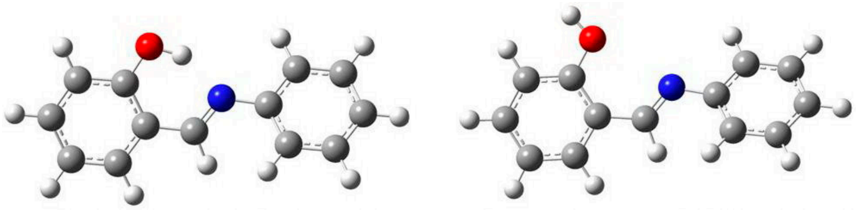

Enol-form (0.0 kcal/mol) **Open-form (13.79 kcal/mol)**

Figure S2. Computed energies of different conformers of **2** are specified relative to the Enol-form (DFT/B3LYP/6-31G **).

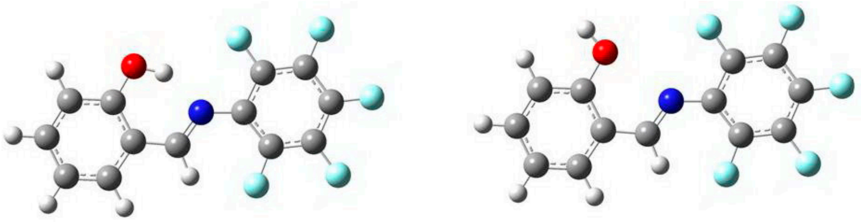

Enol-form (0.0 kcal/mol) **Open-form (12.27 kcal/mol)**

Figure S3. Computed energies of different conformers of **3** are specified relative to the Enol-form (DFT/B3LYP/6-31G **).

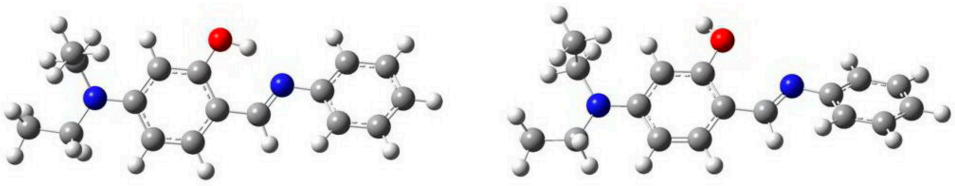

Enol-form (0.0 kcal/mol) **Open-form (14.35 kcal/mol)**

Figure S4. Computed energies of different conformers of **4** are specified relative to the Enol-form (DFT/B3LYP/6-31G**).

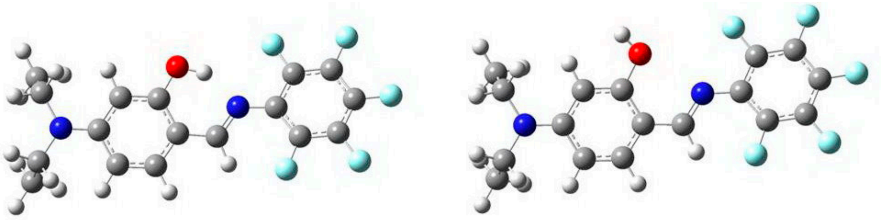

Enol-form (0.0 kcal/mol) **Open-form (12.50 kcal/mol)**

Figure S5. Frontier molecular orbitals of compound **4** in the ground and excited state.

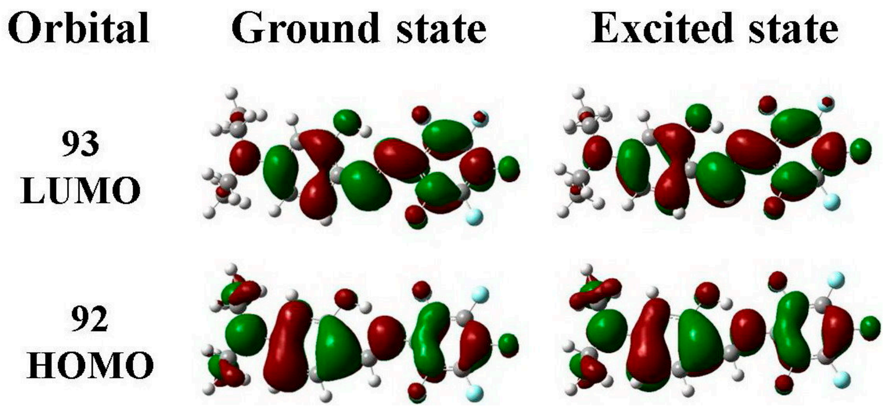

Figure S6. DFT geometry-optimized structure of **4**. The blue arrows represent the molecules' dipole orientations.

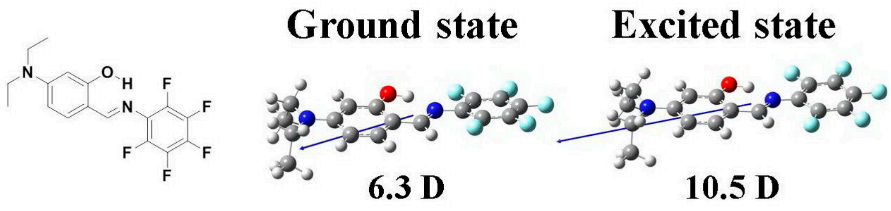

Figure S7. ^{1}H NMR of **4**.

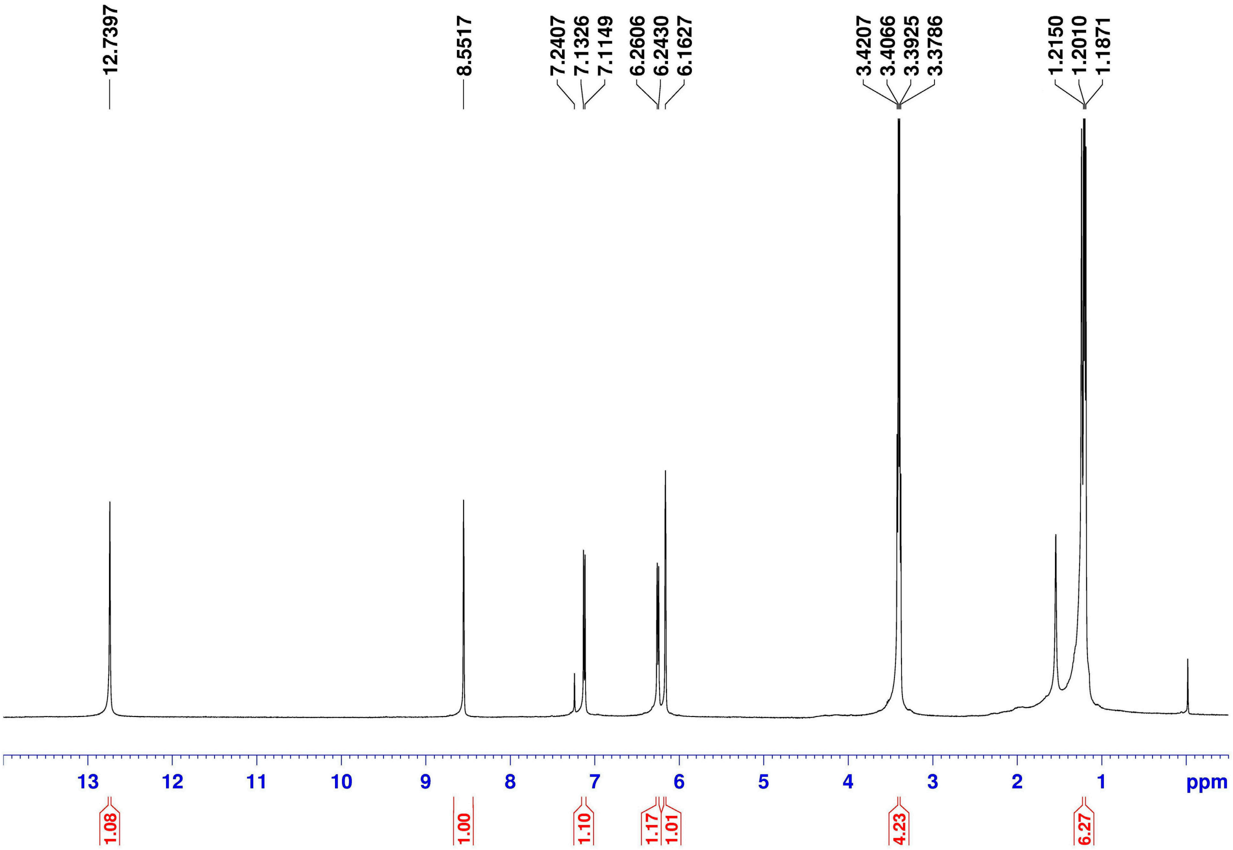

Figure S8. ^{13}C NMR of **4**.

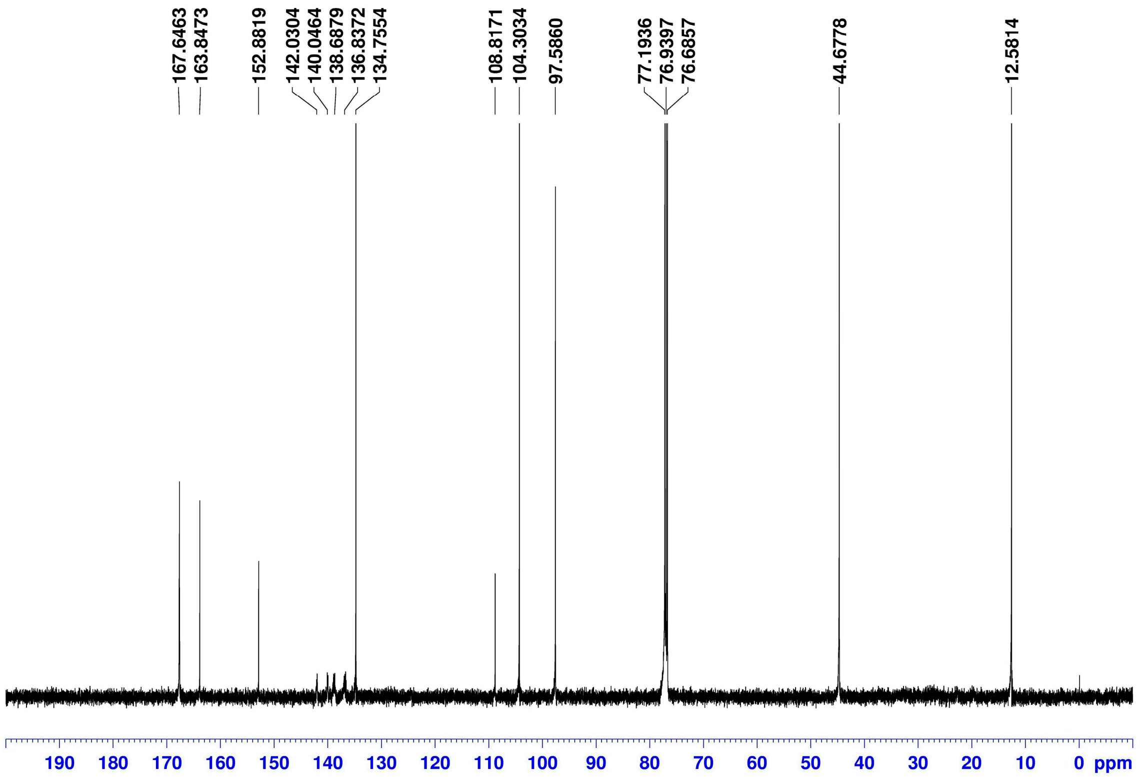

Table S1. The converged geometries of compounds **1**–**4**.

Compound 1	X	Y	Z
C	5.79143	1.63774	−0.49322
C	4.41101	1.58953	−0.57784
C	3.69115	0.44067	−0.18246
C	4.41066	−0.6883	0.30683
C	5.81068	−0.63045	0.38738
C	6.48546	0.51683	−0.00621
H	6.33162	2.52801	−0.79883
H	3.85496	2.44618	−0.95257
H	6.33902	−1.50074	0.7629
C	2.25142	0.4323	−0.28528
H	1.7847	1.34215	−0.68412
N	1.53228	−0.5944	0.04776
C	0.13603	−0.55037	−0.00119
C	−0.55044	−1.73717	−0.31563
C	−0.61452	0.60583	0.29715
C	−1.93333	−1.75043	−0.3734
H	0.02429	−2.63372	−0.52515
C	−1.99907	0.58915	0.24576
H	−0.10482	1.51404	0.60364
C	−2.69621	−0.59095	−0.10025
H	−2.44696	−2.67322	−0.63089
H	−2.53999	1.49554	0.48745

Table S1. *Cont.*

Compound 1	X	Y	Z
O	3.79603	−1.81464	0.69398
H	2.81787	−1.6851	0.56081
H	7.56963	0.54463	0.06382
H	−3.76032	−0.68388	−0.16308
C	5.79143	1.63774	−0.49322
C	4.41101	1.58953	−0.57784
C	3.69115	0.44067	−0.18246
C	4.41066	−0.6883	0.30683
C	5.81068	−0.63045	0.38738
C	6.48546	0.51683	−0.00621
H	6.33162	2.52801	−0.79883
H	3.85496	2.44618	−0.95257
H	6.33902	−1.50074	0.7629
C	2.25142	0.4323	−0.28528
H	1.7847	1.34215	−0.68412
N	1.53228	−0.5944	0.04776
C	0.13603	−0.55037	−0.00119
C	−0.55044	−1.73717	−0.31563
C	−0.61452	0.60583	0.29715
C	−1.93333	−1.75043	−0.3734
H	0.02429	−2.63372	−0.52515
C	−1.99907	0.58915	0.24576
H	−0.10482	1.51404	0.60364
C	−2.69621	−0.59095	−0.10025
H	−2.44696	−2.67322	−0.63089
H	−2.53999	1.49554	0.48745
O	3.79603	−1.81464	0.69398
H	2.81787	−1.6851	0.56081
H	7.56963	0.54463	0.06382
H	−3.76032	−0.68388	−0.16308
**Compound 2**	**X**	**Y**	**Z**
C	−3.03573	−0.17553	−1.53526
C	−2.00755	−1.03433	−1.12387
C	−1.29866	−0.76455	0.05452
C	−1.61795	0.36402	0.82153
C	−2.64613	1.22282	0.41014
C	−3.35502	0.95304	−0.76826
H	−3.57698	−0.38151	−2.43499
H	−1.76377	−1.89602	−1.7095
H	−2.88991	2.08451	0.99576
H	−4.14005	1.60875	−1.08236
C	−0.1688	−1.70828	0.5066
H	0.07498	−2.56998	−0.07902
N	0.48556	−1.45926	1.59435

Table S1. *Cont.*

Compound 2	X	Y	Z
C	1.56407	−2.36009	2.02588
C	1.88336	−3.48867	1.25888
C	2.27296	−2.09032	3.20428
C	2.91153	−4.34747	1.67027
C	3.30113	−2.94912	3.61567
C	3.62042	−4.07769	2.84867
O	−0.89459	0.63931	2.02397
H	0.00121	0.30392	1.94242
F	1.96538	−1.00314	3.94315
F	3.98402	−2.68924	4.75085
F	4.61089	−4.90499	3.24497
F	3.21911	−5.43465	0.9314
F	1.20047	−3.74855	0.1237
**Compound 3**	**X**	**Y**	**Z**
C	3.87909	1.4428	−0.47129
C	2.50356	1.45192	−0.5212
C	1.72622	0.29252	−0.30898
C	2.43049	−0.92056	−0.03379
C	3.82107	−0.9364	0.01961
C	4.58757	0.22832	−0.20983
H	4.4082	2.37281	−0.62306
H	1.98966	2.38963	−0.72258
H	4.27784	−1.89023	0.24458
C	0.29927	0.35111	−0.36431
H	−0.14115	1.33023	−0.59256
N	−0.45775	−0.69662	−0.18617
C	−1.8454	−0.59666	−0.16983
C	−2.59204	−1.71942	−0.57991
C	−2.5474	0.54508	0.27898
C	−3.97431	−1.68219	−0.58198
H	−2.05839	−2.60606	−0.90748
C	−3.93121	0.57921	0.28093
H	−1.99703	1.39957	0.66025
C	−4.68599	−0.53396	−0.15842
H	−4.5306	−2.55505	−0.91486
H	−4.42958	1.47174	0.6386
O	1.7793	−2.07395	0.18196
H	0.80359	−1.88495	0.09698
N	5.96796	0.20064	−0.19455
C	6.70253	1.46979	−0.33101
H	6.49284	2.12349	0.53042
H	6.31444	1.98467	−1.21623
C	8.21529	1.34171	−0.5023
H	8.62443	2.34368	−0.6697

Table S1. *Cont.*

Compound 3	X	Y	Z
H	8.71193	0.93049	0.38191
H	8.47755	0.72719	−1.36993
C	6.67191	−0.98834	0.2932
H	7.68819	−0.96499	−0.10169
H	6.20828	−1.87233	−0.15266
C	6.71013	−1.11541	1.8219
H	5.70054	−1.15309	2.24258
H	7.2368	−2.03163	2.11311
H	7.23382	−0.26604	2.2751
H	−5.7544	−0.58422	−0.188
**Compound 4**	**X**	**Y**	**Z**
C	4.66125	−1.38678	0.33741
C	3.27011	−1.55534	0.32229
C	2.43201	−0.44748	0.13746
C	2.98506	0.82894	−0.03225
C	4.3762	0.99751	−0.01713
C	5.2143	−0.11035	0.1677
H	5.30115	−2.23265	0.47853
H	2.84784	−2.52992	0.45186
H	4.79846	1.97209	−0.14671
O	2.12986	1.95941	−0.22086
H	1.3234	1.68072	−0.66081
C	0.90328	−0.63272	0.12084
H	0.48102	−1.60729	0.25042
N	0.12965	0.38992	−0.04977
C	−1.32959	0.21311	−0.06563
C	−1.88263	−1.06331	0.10409
C	−2.16768	1.32097	−0.25046
C	−3.27378	−1.23188	0.08897
C	−3.55883	1.15241	−0.26558
C	−4.11187	−0.12402	−0.09586
N	6.67354	0.06646	0.18356
C	7.19899	−0.07379	−1.18214
H	6.96174	−1.04736	−1.55734
H	6.75655	0.66898	−1.81256
C	8.72772	0.11144	−1.16552
H	9.17016	−0.63133	−0.5351
H	9.11019	0.00935	−2.1596
H	8.96497	1.08501	−0.79031
C	7.28138	−0.95398	1.04965
H	7.04413	−1.92755	0.67445
H	6.89891	−0.85189	2.04373
C	8.81011	−0.76875	1.06627
H	9.25256	−1.51152	1.69669

Table S1. *Cont.*

**Compound 4**	**X**	**Y**	**Z**
H	9.19258	−0.87084	0.07219
H	9.04736	0.20482	1.44148
F	−1.63492	2.55058	−0.41395
F	−4.36618	2.21963	−0.44363
F	−5.45199	−0.2864	−0.11042
F	−3.80654	−2.46148	0.25246
F	−1.07528	−2.13054	0.28214
